# Supplementary material for: Exploring antecedents and outcomes of burnout among emergency department staff using the job demands-resources model: A scoping review protocol
Source: PLoS One. 2024 Mar 14;19(3):e0300040. doi: 10.1371/journal.pone.0300040 (PMC10939206; doi:10.1371/journal.pone.0300040)
Supplement: Attachment — Submitted filename: Response to Reviewers.docx [file pone.0300040.s003.docx]

Dear Editor and Reviewers,

On behalf of the co-authors, we express our gratitude for the opportunity to revise our manuscript. We sincerely appreciate your insightful comments and suggestions on our manuscript titled "Exploring antecedents and outcomes of burnout among emergency department staff using the Job Demands-Resources model: A scoping review protocol" (No.: PONE-D-23-25522 ). We have thoroughly reviewed and diligently revised our manuscript following the reviewers' comments. The reviewer's comments are below in italicized font, and specific concerns have been numbered. Notably, our responses are in normal font, while additions to the manuscript are highlighted in green text. Moreover, all changes/additions to the latest manuscript use Microsoft Word's Track changes method. Below is a point-by-point response to the Editor and Reviewers' comments and concerns.

**Responses to the comments of the Editor:**

***Comment 1:*** *Please ensure that your manuscript meets PLOS ONE's style requirements, including those for file naming.*

**Response**: Thank you for your reminder. We have thoroughly reviewed and revised the manuscript style and file names in accordance with the journal's style requirements.

***Comment 2:*** *We noticed you have some minor occurrence of overlapping text with the following previous publication(s), which needs to be addressed:-* [*https://doi.org/10.1111/1742-6723.12135*](https://doi.org/10.1111/1742-6723.12135)*. In your revision ensure you cite all your sources (including your own works), and quote or rephrase any duplicated text outside the methods section.*

**Response:** In response to these concerns, we have carefully rephrased the duplicated text and cited all sources involved(Lines 58~64).

***Comment 3:*** *PLOS requires an ORCID iD for the corresponding author in Editorial Manager on papers submitted after December 6, 2016. Please ensure that you have an ORCID iD and that it is validated in Editorial Manager.*

**Response:** In compliance with this requirement, I have updated my information and linked my ORCID iD with the Editorial Manager system for our submission.

***Comment 4:*** *Please review your reference list to ensure that it is complete and correct.*

**Response:** Thank you for your reminder. We have examined each citation in our manuscript to ensure the reference list is complete and correct.

**Responses to additional Editor comments:**

***Comment:*** *Information regarding burnout could be found in articile Who Cares What the Doctor Feels: The Responsibility of Health Politics for Burnout in the Pandemic. Kindly review and evaluate the requested work to determine whether they are relevant and should be cited. Once you have completed the revisions, please submit the revised version of your manuscript through our online submission system. Include both a clean version and a tracked changes version to aid our review process.*

**Response:** Thank you for your valuable feedback and for providing the reference. We have carefully reviewed the article to assess its relevance to our work and cited this article in our revised manuscript, ensuring that it supports our findings(Lines 55~57).

As instructed, we are submitting a clean version and a tracked changes version of our revised manuscript through the online submission system.

**Responses to the comments of Reviewer** #1**:**

***Comment:*** *The manuscript appears to be well structured, overall. A suggested minor edit is to offer a concise overview of the anticipated benefits and implications of the scoping review in the Introduction section. Explain how the review's outcomes could affect healthcare organizations or practitioners. This background can assist readers grasp the significance of your findings.*

**Response:** Thank you for your suggestion. We've revised the Introduction to include a concise overview of our scoping review's anticipated benefits and implications (Lines 92~98).

**Responses to the comments of Reviewer** #2**:**

***Comment 1:*** *Introduction and Rationale: The Introduction clearly states the high prevalence of burnout among ED staff due to chronic job stress. However, it would benefit from more specific data or statistics to emphasize the magnitude of the problem. Also, it would be useful to briefly mention why the Job Demands-Resources (JD-R) model is chosen for this study.*

**Response:** Thank you for your insightful feedback. We have supplemented the Introduction with additional specific data and statistics to highlight the prevalence of burnout among ED staff (Lines 49~55).

We've also briefly explained why the Job Demands-Resources (JD-R) model is particularly relevant and suitable for this study (Lines 68~72).

***Comment 2:*** *Methodology: The methodology section is thorough, explaining the use of the Joanna Briggs Institute methodology and the PRISMA-ScR checklist. However, it would be beneficial to include more detailed information on the criteria for selecting studies (e.g., year range, type of studies included).*

**Response:** Thank you for your kind reminder. As suggested, we have updated the methodology section to include more detailed information on our study selection criteria. Specifically, we have clarified that the included study types are original articles employing quantitative and qualitative research methods. We have also defined the year range of the studies from inception to August 19, 2023(Lines 134~136).

We thank you again for giving us an opportunity to revise this manuscript and look forward to hearing from you.

Dr. Anita

Corresponding author

University Putra Malaysia
